# Job Competency and Intention to Stay among Nursing Assistants: The Mediating Effects of Intrinsic and Extrinsic Job Satisfaction

**DOI:** 10.3390/ijerph18126436

**Published:** 2021-06-14

**Authors:** Yu-Chia Chang, Te-Feng Yeh, I-Ju Lai, Cheng-Chia Yang

**Affiliations:** 1 Department of Healthcare Administration, Asia University, Taichung 41354, Taiwan; ycchang@asia.edu.tw; 2 Department of Medical Research, China Medical University Hospital, China Medical University, Taichung 40433, Taiwan; 3 Department of Healthcare Administration, Central Taiwan University of Science and Technology, Taichung 40601, Taiwan; tfyeh@ctust.edu.tw; 4 Department of Nursing, Taichung Veterans General Hospital, Taichung 40705, Taiwan; sam50526@vghtc.gov.tw

**Keywords:** job competency, intention to stay, intrinsic job satisfaction, extrinsic job satisfaction, nursing assistants

## Abstract

This study investigated the influences of nursing assistants’ job competency on their intrinsic and extrinsic satisfaction and intention to stay in the profession of long-term care institutions. Understanding the relationship between job competency and job satisfaction, both intrinsic and extrinsic, would enable institutions to strengthen service workers’ intention to stay and to retain essential personnel. This study was a cross-sectional study in which nursing assistants from 26 nursing homes and 15 elderly welfare institutions in Taiwan. The relationship between job competency and intention to stay was discovered to be significantly mediated by intrinsic and extrinsic job satisfaction. Given the staff shortages and difficulty retaining staff in long-term care environments, organizations must be able to strengthen employees’ intention to stay; one suggestion is to improve the employees’ competency, because higher competency results in higher quality of care and greater extrinsic job satisfaction. Furthermore, greater job competency is more likely to result in affirmation and accomplishment, both of which increase intrinsic job satisfaction and thus positively influence intention to stay.

## 1. Introduction

Although Taiwan implemented a 10-year long-term care project (2007–2016) in which a comprehensive community care model was developed, the project experienced nursing assistant shortages and a weak intention to stay in long-term care services among employees [1]. At present, nursing assistants are on the front line of long-term care and have wide-ranging responsibilities for daily living care. They have become the people with whom care recipients have their most intimate relationship. Consequently, their professional performance directly affects the quality of life of older people, and measuring nursing assistants’ intention to stay is thus a crucial topic.

Many studies discuss the intention to stay of nursing assistants, which is mainly related to job satisfaction [2,3,4,5]. Although many studies have explored factors affecting the job satisfaction of nursing assistants (e.g., job stress [2], psychological empowerment [6], and received support from peers and managers [6], these studies did not consider the personal abilities of nursing assistants. Studies have revealed that employees with greater “demands–abilities fit” can more efficiently complete the tasks assigned by their organizations [7], thereby achieving greater job satisfaction [8]. Nursing employees with higher job competency can exercise their abilities more confidently when completing nursing tasks, which subsequently affects their job satisfaction and intention to stay. A few studies related to job satisfaction have set job satisfaction as a single-dimension variable in their analyses [9,10]. However, Tsounis and Sarafis contended that job satisfaction is a multi-dimensional variable [11]. This study sought to validate the mediating effects of intrinsic and extrinsic satisfaction on job competency and intention to stay. The aim is to increase nursing assistants’ intention to stay in their position through greater understanding of their job competency and job satisfaction—intrinsic and extrinsic—thereby enabling retention of essential talents in Taiwan’s long-term care industry. After reviewing important literature, the research hypotheses of this study were formulated.

### 1.1. Relationship between Job Competency to Intention to Stay

Job competency refers to the knowledge, attitude, and skills that employees must have to perform their work. These competencies can be evaluated and can be improved through training [12]. Any discussion of job competency must involve the concept of person–job fit. According to Edwards, person–job fit can be divided into the fit between demands and abilities—how the employee’s abilities and knowledge meet the work requirements—and that between need and supplies—whether the salary compensation and sense of achievement provided by the job satisfy the employee’s needs [13].

Relevant studies have shown that person–job fit is negatively associated with turnover intention [14]. Organizations must pay greater attention to person–job fit to increase employees’ level of engagement and decrease voluntary turnover rate [15]. Thus, employees who identify as having a higher job competency regarding their work gain more resources (e.g., work-related skills) and are more hesitant to consider leaving their jobs [5]. Based on the above arguments, we formed an initial expectation of a positive association between nurse job competency and nursing assistants’ intention to stay.

### 1.2. Extrinsic Satisfaction and Intrinsic Satisfaction

Job satisfaction reflects how employees feel about their current work duties; it is the emotional response generated by the role that the employee plays in the organization [16]. Motivation-hygiene theory states that humans have two types of basic needs [17]. One type is motivator needs, otherwise known as intrinsic factors, which are relevant to the work itself and can be satisfied on the job, because they represent an employee’s psychological needs and prompt long-term impetus [18]. By contrast, hygiene factors are influenced by the external environment and can be considered extrinsic factors. These factors are related to the work environment and include the workplace environment, channels of promotion, salary, support from managers, and rapport with coworkers.

In this study, job satisfaction was distinguished into being from intrinsic and extrinsic factors. Intrinsic factor satisfaction refers to satisfaction gained from the actual work, such as a sense of achievement, a sense of responsibility, and self-respect; extrinsic factor satisfaction is defined as satisfaction gained from the work environment or organization, such as salary, system of promotion, and leadership. Scholars have stated the influence of extrinsic satisfaction on intrinsic satisfaction [19,20]. Using motivation-hygiene theory, this study posited that when nursing assistants’ basic hygiene needs—salary and benefits—are met, nursing assistants begin pursuing motivator needs—personal growth and a sense of accomplishment. Therefore, the following hypothesis was proposed:

**Hypothesis H1.**  *Extrinsic satisfaction has a positive influence on intrinsic satisfaction.*

### 1.3. Relationship between Job Competency and Job Satisfaction

A widespread belief is that a closer fit between demands and abilities leads to greater job satisfaction. Thus, employees with a closer demands–abilities fit are more likely to be competent at their job and adapt to the job more quickly; they are also less likely to experience work pressure, resulting in greater job satisfaction [21]. According to Peng and Mao, employees with closer demands–abilities fit can more efficiently complete tasks, have less work stress, and are more likely to earn their manager’s recognition and praise [7]. Therefore, they are more confident in their work and have greater self-efficacy and a higher sense of accomplishment. Employees with greater self-efficacy have more confidence when faced with challenges in their work, leading to greater job satisfaction [8].

The most direct result of closer demands–abilities fit in an employee is higher work performance. This is because organizations typically reward high performers rather than low performers [22,23]. One study argued that being able to meet demands to merit rewards—such as bonuses and promotion—is dependent on whether the individual’s work meets the conditions of demands–abilities fit [21]. More competent nursing assistants have higher work performance, which may lead to higher compensation, more promotion opportunities, increased welfare, or recognition from superiors. These benefits then affect the job satisfaction of employees in their work environment or organization. The following two hypotheses were proposed in this study:

**Hypothesis H2.**  *Higher job competency leads to greater intrinsic satisfaction.*

**Hypothesis H3.**  *Higher job competency leads to greater extrinsic satisfaction.*

### 1.4. Relationship between Job Satisfaction and Intention to Stay

Intention to stay refers to an employee’s intention either to remain in their current job or to resign [24]. It reflects the likely behavior of employees who are carefully considering their options. Job satisfaction is considered the most decisive factor influencing intention to stay, and job satisfaction and intention to stay are strongly positively correlated [25,26].

Salary and benefits are relevant to job satisfaction and influence intention to resign [27]. Although extrinsic satisfaction is crucial to an employee’s intention to stay, research into job satisfaction has discovered that the intrinsic value of work has a similar influence on intention to stay as extrinsic satisfaction. All employees hope to be respected in an organization and be more than a simple laborer; therefore, when an individual thinks that their colleagues or manager finds them valuable, their self-esteem increases [28], and when an individual believes themselves to have value in and to have made contributions to the work environment, they have stronger intention to stay [29].

In summary, if an organization can create a supportive work environment, satisfactory salary, and benefits, it can strengthen employees’ intention to stay [30]. This study hypothesized that the intrinsic and extrinsic satisfaction that nursing assistants obtain from their job—such as a sense of accomplishment, a sense of responsibility, esteem, salary, and promotions—influence employees’ intention to stay. The following two hypotheses were thus made:

**Hypothesis H4.**  *Higher intrinsic satisfaction results in stronger intention to stay.*

**Hypothesis H5.**  *Higher extrinsic satisfaction results in stronger intention to stay.*

### 1.5. Job Satisfaction as a Mediator between Job Competency and Intention to Stay

Porter, Bigley, and Steers stated that employees receive compensation and rewards for outstanding work performance, and these rewards increase the employee’s job satisfaction [31]. When employees are satisfied, their intention to stay is stronger [25]. Morley et al. discovered that poor fit between an individual and organization leads to job dissatisfaction, influencing intention to stay [32]. Job satisfaction has been proven to have a mediating effect on the relationship between person–job fit and intention to resign. When the person–organization fit is closer and the compatibility between needs and abilities is high, the person tends to have a more positive work attitude and more positive behavior [22,23]. A person with abilities highly compatible with their organization’s needs is a highly competent employee with high work performance. This results in high job satisfaction for the employee from their work environment or organization, as well as possibly a greater salary, a promotion, a sense of accomplishment, a sense of responsibility, or high self-esteem. Increasing these intrinsic and extrinsic factors of satisfaction affects intention to stay. Furthermore, extrinsic satisfaction strongly influences intrinsic satisfaction [19,20]. Therefore, the following hypotheses were proposed in this study:

**Hypothesis H6.**  *Intrinsic satisfaction mediates the relationship between job competency and intention to stay.*

**Hypothesis H7.**  *Extrinsic satisfaction mediates the relationship between job competency and intention to stay.*

**Hypothesis H8.**  *Job competency influences intrinsic satisfaction through extrinsic satisfaction, ultimately influencing intention to stay.*

## 2. Materials and Methods

### 2.1. Sample and Procedure

This study adopted a cross-sectional design. All certificated long-term care institutions in Taichung city, Taiwan were surveyed, including 26 nursing homes and 15 elderly welfare institutions. All nursing assistants from those institutions were interviewed using a self-designed structured questionnaire for a quantitative investigation and analysis. The study was reviewed and approved by the institutional review board (IRB) of the Taichung Jen-Ai Hospital (No: 10817). The researchers visited the institutions to explain the purpose and procedure of the study and emphasized the voluntariness, privacy, and confidentiality of the participants. Participant willingness to participate was determined by whether they were willing to answer the questionnaire and submit their responses to us. Sealed envelopes containing a brief description of the study, the questionnaire, and a return envelope were distributed to the participants, who were required to complete the questionnaire within two weeks and return it using the return envelope. In addition, one NT $100 voucher for 7-Eleven was included in each of the sealed envelopes to increase the participation rate. A total of 383 questionnaires were distributed, and 333 valid questionnaires were recovered, yielding a recovery rate of 87%. The four major components of the questionnaire were personal information, scales on job competency, job satisfaction, and intention to stay.

### 2.2. Job Competency Scale

After an extensive review of the available literature, the job competency scale employed in this study was adapted from the “Long-term care, supports, and services competency model” [33]. The model has five domains: those related to personal effectiveness, academic, workplace, industry-wide technical, and industrial-sector technical competencies. Among them, “industry-wide technical competency” was used to refer to job competency in the long-term care industry and was measured using seven aspects: (1) long-term care, supports, and services; (2) supporting daily living; (3) crisis prevention and conflict resolution; (4) ethics; (5) documentation; (6) laws and regulations; and (7) patient health and safety.

The Aspect 5 and 6 were excluded from the final questionnaire because the respondents of this study were frontline nursing assistants and not managers. The self-designed job competency scale used in this study for frontline nursing assistants consisted of 21 items in five dimensions (Aspects 1 through 4, and Aspect 7) (see Appendix A). Six experts reviewed the questionnaire with a content validity index (CVI) of 0.94. These items were scored using a 5-point Likert scale ranging from 1 (strongly disagree) to 5 (strongly agree) and were combined into a formative measurement construct.

To explore the structure of the job competency scale, an exploratory factor analysis (EFA) was conducted. Five dimensions were identified that explained 72.7% of the variability. As shown in Table 1, the job competency scale and its subscales showed very good internal consistency (job competency scale: Cronbach’s α = 0.87; long-term care, supports, and services: Cronbach’s α = 0.849; supporting daily living: Cronbach’s α = 0.829; crisis prevention and conflict resolution: Cronbach’s α = 0.849; ethics: Cronbach’s α = 0.898; patient health and safety: Cronbach’s α = 0.787).

### 2.3. Job Satisfaction Scale

The Minnesota Satisfaction Questionnaire [34] was referenced and revised to create a measure of job satisfaction. The scale had 12 and 8 items for the intrinsic and extrinsic satisfaction dimensions, respectively. Intrinsic satisfaction refers to the values, sense of responsibility, sense of belonging, and social standing originating from the work itself, whereas extrinsic satisfaction refers to salary and benefits, promotion, on-the-job training, and feelings from interactions with managers and coworkers. The 20 items were scored using a 5-point Likert scale ranging from 1 (strongly disagree) to 5 (strongly agree). The Cronbach’s alpha reliability coefficient of job satisfaction in this sample was 0.923.

### 2.4. Intention to Stay

The scale of Milliman, Gatling, and Kim was referenced and revised to measure whether nursing assistants intended to remain in their job at present and in the long term [24]. The two items were “I currently intend to continue in my work as a nursing assistant” and “I intend to still be a nursing assistant a year from now”; they were scored using a 5-point Likert scale ranging from 1 (strongly disagree) to 5 (strongly agree). The Cronbach’s alpha reliability coefficient of intention to stay in this sample was 0.943.

### 2.5. Reliability and Validity of the Model

The construct of the job competency scale used in this study was first-order reflective, second-order formative. Formative metrics do not need to be measured for internal consistency or reliability [35] but must prevent overly strong correlations among the measurement variables, which prevents overly high collinearity. For this purpose, variance inflation factor (VIF) values were calculated; if VIF > 10, the collinearity was too high. The VIFs of the formative metrics in this study were between 1.55 and 3.07, indicating that the job competency scale did not have any collinearity issues. The VIF values for all items are presented in Table 1. The standardized root-mean-square residual (SRMR) was used to evaluate the fit of the study model. An SRMR for the saturated and estimated models smaller than 0.08 indicated an acceptable model fit [36]. The SRMR for the saturated and estimate models in this study was 0.74, indicating that the models had an acceptable fit. The individual item reliability indicated the factor loading that the measurement variables had on the latent variables and tested each factor loading for statistical significance. All factor loadings in this study were greater than the suggested value of 0.5, indicating significance. The factor loading values of the sample were 0.594–0.974, meeting the threshold suggested by Hair et al. [37] (Table 1).

The measurement model of this study’s reflective indicators was appraised by calculating the individual item reliability, composite reliability (CR), and average variance extracted (AVE). The CR of the latent variables was the composite of the reliability of all the measurement variables and represented the internal consistency of the constructed index. Higher reliability indicated higher internal consistency of the latent variables. China suggested a CR of 0.7 or greater [38]; Table 1 shows that the CR for each variable in this study was between 0.880 and 0.972, greater than the 0.7 standard, indicating favorable internal consistency. The AVE of the latent variables indicated the power of each measurement variable to explain the latent variable; higher AVE indicated that the latent variable had higher discriminatory validity and convergent validity. Fornell and Larcker suggested that the AVE must be greater than 0.5 [39]; as detailed in Table 1, the AVEs of the latent variables were between 0.588 and 0.710, all greater than the 0.5 standard value, indicating that the reflective measurement variables had favorable convergent validity.

Lastly, discrimination validity was measured by calculating the square root of the AVE. If the square root was greater than the other coefficients in the same construct, the relationships among the latent constructs were weaker than the relationships within the construct, indicating that the measurement model had favorable discrimination validity. Because the formative metrics do not require measurement of the square root of the AVE [40], only the square root of the AVE for the reflective metrics was measured and the matrix compared. This study was greater than the coefficients of every dimension (Table 2). Therefore, the dimensions had high discrimination validity.

### 2.6. Data Analysis

The causal model between the latent variables was analyzed using partial least squares (PLS) for constructing predictive models. The PLS method is suitable for simultaneously constructing formative and reflective models for measuring variables; the obtained models are superior to general linear structural relationship models, so PLS is suitable for exploratory research. PLS can accept dimensions with a single item and is not limited by variable allocation or the number of sizes; it has satisfactory predictive and explanatory abilities [41]. In this study, the measurement and structural models were analyzed using SmartPLS. The bootstrap resampling method was then used to draw 5000 samples as parameter calculations and inferences for estimation [42].

## 3. Results

### 3.1. Demographics and Characteristics

The vast majority of the respondents were women (*n* = 281; 84.4%); only 52 of the respondents were men (15.6%). Furthermore, 72 respondents were 30–39 years (21.6%), whereas 71 were 40–49 years (21.3%), 94 were 50–59 years (28.2%), and 43 were 60 years or older (12.9%). The mean age of the respondents was 44.24 (standard deviation = 12.54). Regarding level of education, 68 respondents had a junior high school education or lower (20.4%), 128 had a high school or technical school education (38.4%), and 70 had a junior college education (21%). Regarding marital status, 221 of the nursing assistants were married (66.4%), whereas 112 were unmarried (including divorced or widowed; 33.6%). Most of the respondents were employed in a nursing home (236 respondents, 70.9%), but 97 worked in an elderly welfare institution (29.1%). Regarding work experience, 86 respondents had 5–10 years of experience (25.8%), and 109 respondents had 10 or more years of experience (21.7%). The average number of years of experience was 7.5 years. The average number of cases per respondent was 12.74, and the average number of daily work hours was 9.18 hours. 

To prevent and mitigate CMV problems, pretest prevention and post-test detection were employed in this study [43]. The pretest prevention involved respondents completing the questionnaire anonymously. For the post-test detection, Harman’s single factor test was used to extract six factors with eigenvalues greater than 1 under unrotated circumstances; the cumulative explained variance was 64.6%, and the explained variation of the first factor was 40.01%, which was smaller than 50%. Therefore, the preliminary determination was that CMV had little effect.

### 3.2. Mediation Regression Models of Study Variables

**Figure 1:**
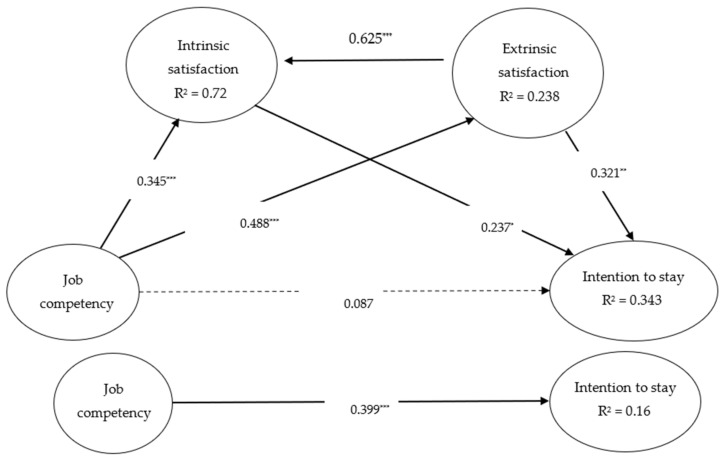
Theoretical model. Note: * *p* < 0.05; ** *p* < 0.01; *** *p* < 0.001.

Using PLS to estimate the path relationships for each dimension, the path values are represented using standardized coefficients, which are detailed in Figure 1. Extrinsic satisfaction had a positive effect on intrinsic satisfaction (β = 0.625, *t* = 17.204, *p* < 0.001); therefore; H1 was supported. Nursing assistants’ job competency also had a positive effect on their intrinsic satisfaction (β = 0.345, *t* = 8.995, *p* < 0.001), indicating that H2 was supported. This study thus found that greater job competency led to higher intrinsic satisfaction. Together, these effects explained 72% of the variance in intrinsic satisfaction. Nursing assistants’ job competency had a positive effect on extrinsic satisfaction (β = 0.488, *t* = 10.733, *p* < 0.001); therefore, H3 was supported. Greater job competency led to higher intrinsic satisfaction. This effect explained 23.8% of the variance in extrinsic satisfaction. Nursing assistants’ intrinsic satisfaction had a positive effect on their intention to stay (β = 0.237, *t* = 2.104, *p* < 0.05); therefore, H4 was supported. Nursing assistants’ extrinsic satisfaction had a positive effect on their intention to stay (β = 0.321, *t* = 3.303, *p* < 0.01); therefore, H5 was supported.

The study used Hair et al.’s, (2016) steps to apply Preacher and Hayes’ approach to the mediation model. First, the study confirmed the direct effect between job competency and intention to stay. This effect was positive and significant (β = 0.399, *t* = 7.502; *p* < 0.001; Figure 1). The second step consisted of including the effect of the mediator variable (intrinsic and extrinsic satisfaction). The indirect effect was positive and significant (H2, H3, H4, and H5 were supported; Figure 1). The mediating effect completely suppressed the direct effect, because the direct relationship between job competency and intention to stay had a β = 0.087, *t* = 1.106; *p* > 0.05, thus producing mediation.

The study analyzed the indirect effects using the bootstrap procedure described (Hair et al., 2016). If the 95% CI of the mediation effect did not contain 0, the mediation effect was significant—that is, a mediation effect existed. The effect of job competency on intention to stay through intrinsic satisfaction was 0.082 (standard error (SE) = 0.074, 95% CI (0.009, 0.158)). The effect of job competency on intention to stay through extrinsic satisfaction was 0.157 (standard error (SE) = 0.048, 95% CI (0.065, 0.256)). The indirect effect on intention to stay from job competency influencing intrinsic satisfaction through extrinsic satisfaction was 0.072 (SE = 0.037, 95% CI (0.006, 0.150)). The three paths did not contain zero, indicating that mediation effects existed, and H6–H8 were supported (Table 3).

## 4. Discussion

Studies on job satisfaction have typically analyzed job satisfaction using a single dimension [9,10]. Tsounis and Sarafis (2018) stated that job satisfaction is a multidimensional concept, and the present study discovered that extrinsic job satisfaction directly influences intrinsic job satisfaction. This signifies that the external factors affecting satisfaction—such as salary—influence the satisfaction employees gain from internal factors affecting satisfaction—such as a sense of accomplishment, a sense of responsibility, and self-esteem. A similar conclusion was made by another study [20]. Furthermore, this study verified the relationships among job competency and intrinsic and extrinsic job satisfaction, discovering that more competent employees are more likely to experience high job satisfaction. This matches the findings of several studies [21]. Scholars have argued that closer person–job fit is linked to higher job competency and job satisfaction. Employees’ job satisfaction influences their intention to stay in their job; this has been verified in many studies [30,44]. The present study also verified the influences of intrinsic and external satisfaction on intention to stay.

Most critically, this study discovered that the relationship between employees’ job competency and intention to stay was subject to the mediating influences of intrinsic and extrinsic job satisfaction. Morley et al. supported this conclusion in their study [32], arguing that when individuals’ job competencies are compatible with the organization’s needs, the individuals display a positive work attitude and receive compensation and a sense of accomplishment due to their high work performance; consequently, the individuals have greater job satisfaction and stronger intention to stay. The relationship between an individual’s job competency and intention to resign is not a direct relationship; more competent employees have stronger intention to stay providing they are satisfied with their job. Similar conclusions were drawn in another study [45].

Recent studies have shown that long-term care workers with higher job competency may pursue career development by resigning from their current jobs [5] because of the gap that exists between their job competency and job satisfaction. This study extended the above concept and explored whether job satisfaction mediated the relationships between ability and intention to stay. The study results verified said mediation effects; that is, for employees with a closer person–job fit and higher job competency, the organizations can offer them salaries commensurate with their performance, and assign them tasks to meet their satisfaction for self-growth, thereby increasing their intention to stay.

Unlike other studies, this study found that job satisfaction has a mediating influence on the relationship between job competency and intention to stay, with extrinsic satisfaction having the strongest effect. This indicates that more competent employees prioritize their extrinsic satisfaction and increase their intrinsic satisfaction through extrinsic satisfaction, ultimately influencing their intention to stay. This is the principal conclusion of this study, and it has not been posited by any other study. In the argument of Maslow that human behavior is caused by needs not being met, the hierarchy of needs must be addressed starting from the lowest level—the deficiency needs—and progress to the highest level—growth needs [46]. This study verified that job satisfaction needs are met starting from the most basic hygiene factors, and when the lowest level of deficiency needs are met, growth needs become important. An employee’s job competency affects whether they receive a decent salary and thus have extrinsic job satisfaction; when extrinsic satisfaction is met, the individual’s sense of accomplishment and confidence in their work rises, increasing their intrinsic satisfaction and ultimately strengthening their intention to stay.

Retaining care staff is vital to long-term care institutions. To address staff shortages and staff being unlikely to remain in the long-term care sector, organizations should first improve the competency of employees. More competent employees can provide higher quality care, and organizations should be willing to provide attractive benefits that increase employees’ extrinsic satisfaction. Furthermore, greater job competency leads to more recognition and approval from residents or family members and, therefore, a sense of accomplishment, increasing intrinsic satisfaction and favorably influencing intention to stay.

Therefore, two suggestions are made herein. Shaheen et al. reported that increasing individuals’ professional effectiveness significantly influences the individuals’ sense of accomplishment and job satisfaction [47], and strategies that take advantage of this fact and improve job competency through training have been proven to help nursing assistants manage problematic behaviors relating to dementia, consequently increasing their job satisfaction [48]. Therefore, training that strengthens nursing assistants’ job competencies to meet an organization’s staffing needs is key to strengthening intention to stay [49,50].

Second, nursing assistants are compensated less well than those working in other medical industries. Typically, people in this profession have lower socioeconomic status, come from single-parent families, or are from an ethnic minority [51]. Furthermore, the stereotype of this type of work having a poor professional image, involving overly long work hours, and causing excessive stress is widespread in society (Chien, 2019). Therefore, increases in nursing assistants’ extrinsic satisfaction, such as their salary, are urgently required; government agencies and long-term care facilities should establish systems for advancing or grading nursing assistants’ competencies. Scholars reported that nursing assistants have less favorable opportunities and channels of promotion than those working in other sectors [52]. Therefore, if nursing assistants’ competencies could be graded or a standard for advancement could be established, nursing assistants could continue to improve their professional abilities and facilities could employ the competency grading to decide upon salaries and compensation for different types of work and increase nursing assistants’ extrinsic satisfaction. Lastly, organizations should take action to increase intrinsic job satisfaction, such as by providing greater psychological empowerment [53], increasing work autonomy [54], or increasing individuals’ sense of accomplishment [9].

This study was subject to some research limitations. First, the number of respondents in this study was insufficient, and if sufficient resources are available in the future for a more extensive investigation, the study results would be more reliable. The cross-sectional nature of the study is its second main limitation. Although we used PLS to analyze the causal model between the latent variables, it is inappropriate to draw causal conclusions. Future studies should collect and analyze data employing longitudinal designs to provide evidence for reciprocal relations and longitudinal mediation and moderation effects. Finally, the current study discussed the mediating effects on nursing assistants’ job competency and intention to stay using only two factors: intrinsic and extrinsic satisfaction. Aloisio et al. stated that support from leaders [6], work autonomy, organizational slack, and perceived psychological empowerment are strongly correlated with job satisfaction. Furthermore, Park et al. argued that work stress [55], the degree of work centrality, and self-efficacy have significant influences on long-term nursing assistants’ job satisfaction. Future studies are suggested to further consider using these factors to investigate the moderating or mediating effects between job competency and intention to stay.

## 5. Conclusions

This study investigated the relationships between job competency and intrinsic and extrinsic job satisfaction. This study discovered that more competent employees have greater job satisfaction and thus a stronger intention to stay. Furthermore, this study found that intrinsic and extrinsic job satisfaction play a mediating role between job competency and intention to stay; more significantly, job competency was found to first influence extrinsic satisfaction and then, through extrinsic satisfaction, influence intrinsic satisfaction to ultimately affect nursing assistants’ intention to stay. Concrete suggestions are provided to long-term care facilities seeking to retain their nursing assistants.

## Figures and Tables

**Table 1:** Reliability and convergent validity of the reflective metrics.

Dimension	Variable	Loading	T-Value	CR	AVE	α Value	VIF
Long-term care support and services	Self1	0.864	53.928	0.898	0.688	0.849	2.489
	Self2	0.828	36.829				2.262
	Self3	0.806	36.015				1.897
	Self4	0.819	33.477				1.912
Support daily living	Sub1	0.723	23.764	0.880	0.595	0.829	1.449
	Sub2	0.801	32.910				1.834
	Sub3	0.790	31.236				1.750
	Sub4	0.752	23.639				1.659
	Sub5	0.787	30.305				1.712
Crisis prevention and conflict resolution	BE1	0.833	35.182	0.888	0.664	0.849	1.944
	BE2	0.865	53.66				2.123
	BE3	0.793	30.116				1.684
	BE4	0.766	22.796				1.568
Ethics	Eth1	0.872	52.974	0.924	0.710	0.898	3.197
	Eth2	0.849	44.485				2.823
	Eth3	0.858	46.891				2.614
	Eth4	0.815	37.159				2.080
	Eth5	0.817	29.521				2.278
Patient health and safety	Heal1	0.849	43.785	0.876	0.702	0.787	1.835
	Heal2	0.861	44.928				1.893
	Heal3	0.801	35.664				1.443
Intrinsic satisfaction	IS1	0.750	28.564	0.945	0.588	0.936	2.442
	IS1	0.760	29.214				2.467
	IS2	0.750	25.273				2.302
	IS3	0.810	41.448				2.627
	IS4	0.733	23.300				2.164
	IS5	0.794	36.666				2.526
	IS6	0.641	17.457				1.679
	IS7	0.722	25.480				1.968
	IS8	0.810	41.314				2.817
	IS9	0.824	43.910				3.036
	IS10	0.835	47.396				3.858
	IS11	0.751	25.156				2.484
	IS12	0.750	28.564				2.442
Extrinsic satisfaction	ES1	0.794	23.752	0.922	0.6	0.902	3.872
	ES2	0.853	44.066				4.613
	ES3	0.844	44.579				3.738
	ES4	0.825	35.895				2.775
	ES5	0.804	32.266				2.572
	ES6	0.594	10.733				1.553
	ES7	0.661	13.385				1.892
	ES8	0.785	31.653				2.209
Intention to stay	Stay1	0.972	192.901	0.972	0.946	0.943	4.931
	Stay2	0.974	213.064				4.931

**Table 2:** Matrix of latent constructs in the measurement model.

Constructs	Mean	SE	1	2	3	4	5	6	7	8	9
1	16.96	2.30	**(0.83)**								
2	21.66	2.43	0.659	**(0.77)**							
3	17.22	2.14	0.531	0.674	**(0.82)**						
4	22.57	2.52	0.519	0.66	0.651	**(0.84)**					
5	12.93	1.61	0.603	0.735	0.672	0.596	**(0.84)**				
6	48.53	6.58	0.546	0.579	0.546	0.471	0.553	**(0.77)**			
7	31.34	4.91	0.403	0.414	0.403	0.35	0.419	0.753	**(0.78)**		
8	91.36	9.26	0.817	0.769	0.817	0.751	0.831	0.663	0.499	NA	
9	8.20	1.77	0.332	0.314	0.332	0.378	0.327	0.549	0.553	0.404	**(0.97)**

Note 1: 1. Long-term care, supports, and services; 2. Supporting daily living; 3. Crisis prevention and conflict resolution; 4. Ethics; 5. Patient health and safety; 6. Intrinsic satisfaction; 7. Extrinsic satisfaction; 8. Job competency; 9. Intention to stay. Note 2: NA indicates that formative metrics do not require measurement of the square root of the AVE. Note 3: The square root of the AVE values shown in bold represent.

**Table 3:** Hypothesis constructs.

Effect	Relations	Estimate	SE	95% CI LL	95% CI UL	Support
Direct						
	H1: ES–IS	0.625 ***	0.036			Yes
	H2: JC–IS	0.345 ***	0.038	0.584	0.715	Yes
	H3: JC–ES	0.488 ***	0.045	0.410	0.584	Yes
	H4: IS–ITS	0.237 *	0.113	0.016	0.434	Yes
	H5: ES–ITS	0.321 **	0.097	0.135	0.497	Yes
Mediating						
	H6: JC–IS–ITS	0.082 *	0.074	0.009	0.158	Yes
	H7: JC–ES–ITS	0.157 ***	0.048	0.065	0.256	Yes
	H8: JC–ES–IS–ITS	0.072 *	0.037	0.006	0.150	Yes

Note 1: * *p* < 0.05; ** *p* < 0.01; *** *p* < 0.001. Note 2: IS: intrinsic satisfaction, ES: extrinsic satisfaction; JC: job competency; ITS: intention to stay.

## Data Availability

The data that support the findings of this study are available on request from the corresponding author. The data are not publicly available due to privacy or ethical restrictions.

## Appendix A

Long-term care support and services

1. I can improve my knowledge of long-term care and continue to learn.
2. I can self-examine and improve the deficiencies for caring for the residents.
3. I participate in professional skills education and training for care services at least twice a year.
4. I will pay attention to long-term care policies and development trends.

Support daily living

1. My care skills are proficient and can ensure the quality of residents’ care.
2. I can help and encourage residents to take in balanced nutrition (including tube-feeding diet).
3. I can assess the psychological condition of the residents and respond in a timely manner.
4. I can provide disease prevention and care for residents.
5. I can help maintain the personal hygiene of the residents.

Crisis prevention and conflict resolution

1. I can build a relationship of trust with residents or family members.
2. I can use communication skills to interact with peers or work partners.
3. I can respect the opinions of my employer.
4. I can express my thoughts appropriately.

Ethics

1. I will not do anything to harm the residents.
2. I will protect the privacy and dignity of the residents.
3. I take my due care responsibilities with all my heart.
4. I abide by work regulations and requirements.
5. I will respect the ideas and wishes of the residents.

Patient health and safety

1. I can assess the changes in the physiological condition of the residents and inform the healthcare personnel if necessary.
2. I will assist in the emergency response and handling of accidents (e.g., fires, natural disasters) to ensure the safety of residents.
3. I can assist in dealing with incidents (e.g., falls, slippage of pipes) and solve the problem correctly.
